# Cytochrome *c* speeds up caspase cascade activation by blocking 14-3-3ε-dependent Apaf-1 inhibition

**DOI:** 10.1038/s41419-018-0408-1

**Published:** 2018-03-06

**Authors:** Carlos A. Elena-Real, Antonio Díaz-Quintana, Katiuska González-Arzola, Adrián Velázquez-Campoy, Mar Orzáez, Abelardo López-Rivas, Sergio Gil-Caballero, Miguel Á. De la Rosa, Irene Díaz-Moreno

**Affiliations:** 1 Instituto de Investigaciones Químicas (IIQ) — Centro de Investigaciones Científicas Isla de la Cartuja (cicCartuja), Universidad de Sevilla – Consejo Superior de Investigaciones Científicas (CSIC), Sevilla, Spain; 2 Institute of Biocomputation and Physics of Complex Systems (BIFI), Joint Unit IQFR-CSIC-BIFI, Universidad de Zaragoza, Zaragoza, Spain; 3 Centro de Investigación Príncipe Felipe, Valencia, Spain; 4 Centro Andaluz de Biología Molecular y Medicina Regenerativa-CABIMER, CSIC-Universidad de Sevilla-Universidad Pablo de Olavide, Sevilla, Spain

## Abstract

Apoptosis is a highly regulated form of programmed cell death, essential to the development and homeostasis of multicellular organisms. Cytochrome *c* is a central figure in the activation of the apoptotic intrinsic pathway, thereby activating the caspase cascade through its interaction with Apaf-1. Our recent studies have revealed 14-3-3ε (a direct inhibitor of Apaf-1) as a cytosolic cytochrome *c* target. Here we explore the cytochrome *c* / 14-3-3ε interaction and show the ability of cytochrome *c* to block 14-3-3ε-mediated Apaf-1 inhibition, thereby unveiling a novel function for cytochrome *c* as an indirect activator of caspase-9/3. We have used calorimetry, NMR spectroscopy, site mutagenesis and computational calculations to provide an insight into the structural features of the cytochrome *c* / 14-3-3ε complex. Overall, these findings suggest an additional cytochrome *c*-mediated mechanism to modulate apoptosome formation, shedding light onto the rigorous apoptotic regulation network.

## Introduction

Apoptosis, a specific form of programmed cell death (PCD), is an essential process for development and homeostasis of multicellular organisms^1^. There are two main apoptotic pathways in mammalian cells: the extrinsic or death receptor-initiated pathway and the intrinsic or mitochondria-dependent pathway^2,3^. The last is triggered by signals, including DNA damage, oxidative stress, and growth factor deprivation, which converge on the release of pro-apoptotic proteins from the mitochondria into the cytosol; one such protein is cytochrome *c* (C*c*). Under homeostasis, C*c* acts as an electron carrier in the mitochondrial respiratory chain^4,5^. Upon its release from mitochondria, C*c* interacts with WD40 domains of the apoptotic protease activating factor-1 (Apaf-1) in the cytosol, enabling the assembly of the apoptosome^6^. This protein platform activates caspase-9 and later caspase-3, initiating the caspase cascade, which executes cell death in an orchestrated way^7,8^.

Recent findings suggest C*c* fulfills multiple roles in apoptosis, beyond Apaf-1 activation and apoptosome assembly. Indeed, C*c* interacts with various cytosolic and nuclear partners along the onset of PCD^9,10^. Hence, the full scope of C*c* role in apoptosis remains un-elucidated. Recently, our group has reported that C*c* inhibits the histone chaperone activity of SET/TAF-Iβ in the nucleus, impairing the formation of core histone-SET/TAF-Iβ complexes under DNA damage^11^. However, the novel functions of cytosolic C*c* stay unveiled, despite a complex network of interactions mediated by C*c* during apoptosis has been suggested^12^.

Therefore, we focused on the interaction between C*c* and protein 14-3-3ε, a novel cytosolic C*c* target under DNA damage^10^. This protein belongs to the 14-3-3 family^13,14^, which comprises seven conserved isoforms (β, γ, ε, η, σ, τ/θ, and ζ), arranged as homo- and heterodimers. Each monomer contains nine α-helices that form a conserved concave groove, used by 14-3-3 proteins to bind their phosphorylated targets^15^ (Supplementary Figure S1). Furthermore, they are also involved in phosphorylation-independent interactions^16–18^. 14-3-3 proteins participate in several cell processes related to metabolism, signal transduction, cell cycle control, apoptosis, transcription, and stress responses^19–24^.

Among 14-3-3ε functions, its ability to inhibit Apaf-1 stands out because it prevents apoptosome assembly and caspase activation^25^. Such inhibition is enhanced by phosphorylation of Apaf-1 at Ser268 by the p90kDa ribosomal S6 kinase-1 (Rsk-1) when the mitogen-activated protein kinases (MAPK) cascade is active. Hence, the interaction of C*c* with 14-3-3ε could modulate such inhibition somehow.

Herein, we show that C*c* hinders 14-3-3ε-mediated Apaf-1 inhibition. Indeed, our results indicate a competition between C*c* and 14-3-3ε for binding to Apaf-1, which enhances caspase activation. Furthermore, this new regulatory mechanism is modulated by phosphorylation of Apaf-1, which enhances its inhibition by 14-3-3ε. We further show that C*c* binds to both the 14-3-3ε concave groove and the convex face, thereby providing a molecular basis for this novel modulation of apoptosome assembly.

## Results

### C*c* interacts with 14-3-3ε in the cytosol under apoptotic conditions

**Fig. 1:**
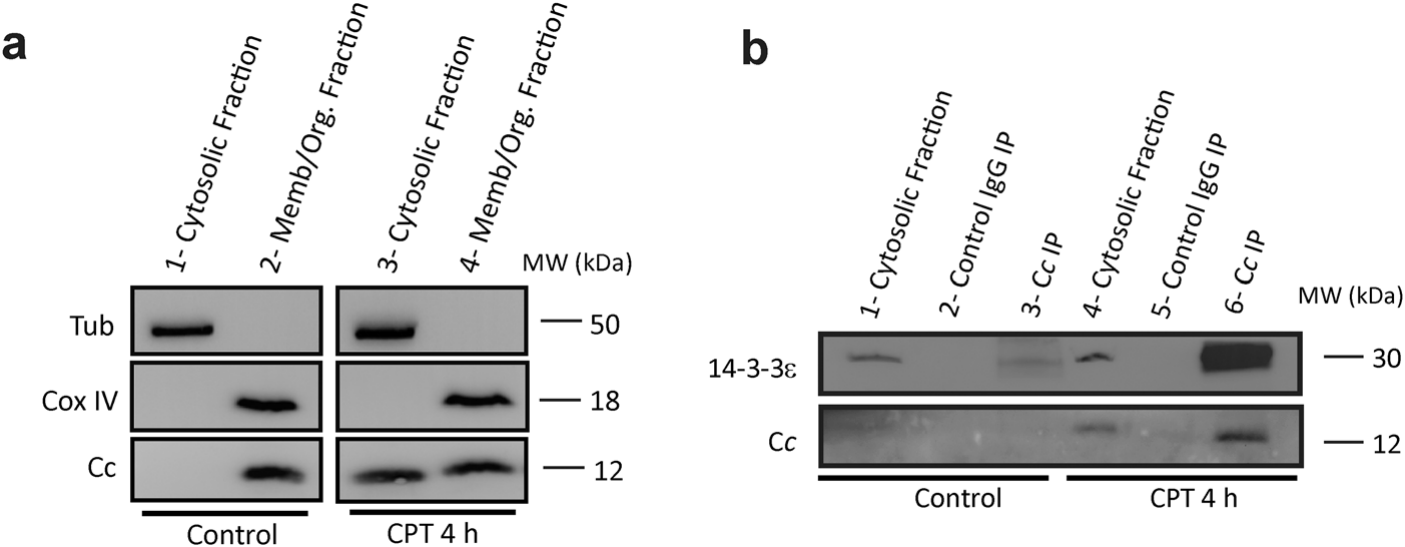
C*c* localization in the cytosol upon cell CPT-treatment. **a** Subcellular fractioning showing the C*c* location upon cell treatment with 20 μM CPT for 4 h. Purity of fractions was verified by western blot using anti-α-Tubulin and anti-CoxIV antibodies for detecting cytosolic and membrane proteins, respectively. **b** C*c*-IP of cytosolic fractions from non-treated (lane 3) and CPT-treated (lane 6) along with the detection of 14-3-3ε by western blot (upper). Mouse IgG was used as control (lanes 2 and 5). Verification of immunoprecipitation of C*c* upon CPT treatment is shown (lower)

To elucidate the extra-mitochondrial function of C*c*, we explored the interaction of C*c* with 14-3-3ε when apoptosis is triggered. To this aim, HeLa cells were treated with the topoisomerase I inhibitor camptothecin (CPT), to induce DNA damage. Then, subcellular fractionation was performed, and C*c* was detected in the cytosol (Fig. 1a, lane 3). However, it remained inside the mitochondria under homeostasis (Fig. 1a, lane 2). The *in cell* C*c* / 14-3-3ε interaction was established as immunoprecipitation (IP) of cytosolic proteins associated with C*c* yielded intrinsic 14-3-3ε in CPT-treated cells (Fig. 1b, lane 6). To further confirm the IP specificity, untreated and CPT-treated cytosolic lysates were probed with a 14-3-3ε antibody (Fig. 1b, lanes 1 and 4, respectively). Negative controls using IgG (Fig. 1b, lanes 2 and 5) did not display any band. Immunoblotting against the anti-C*c* antibody confirmed C*c* IP (Fig. 1b, lane 6).

### C*c* blocks 14-3-3ε-mediated caspase inhibition

Following its release into the cytosol, C*c* targets Apaf-1 to assemble the apoptosome^6^. As 14-3-3ε binds Apaf-1 to prevent caspase activation^25^, we investigated whether C*c* modulates Apaf-1 inhibition by 14-3-3ε.

**Fig. 2:**
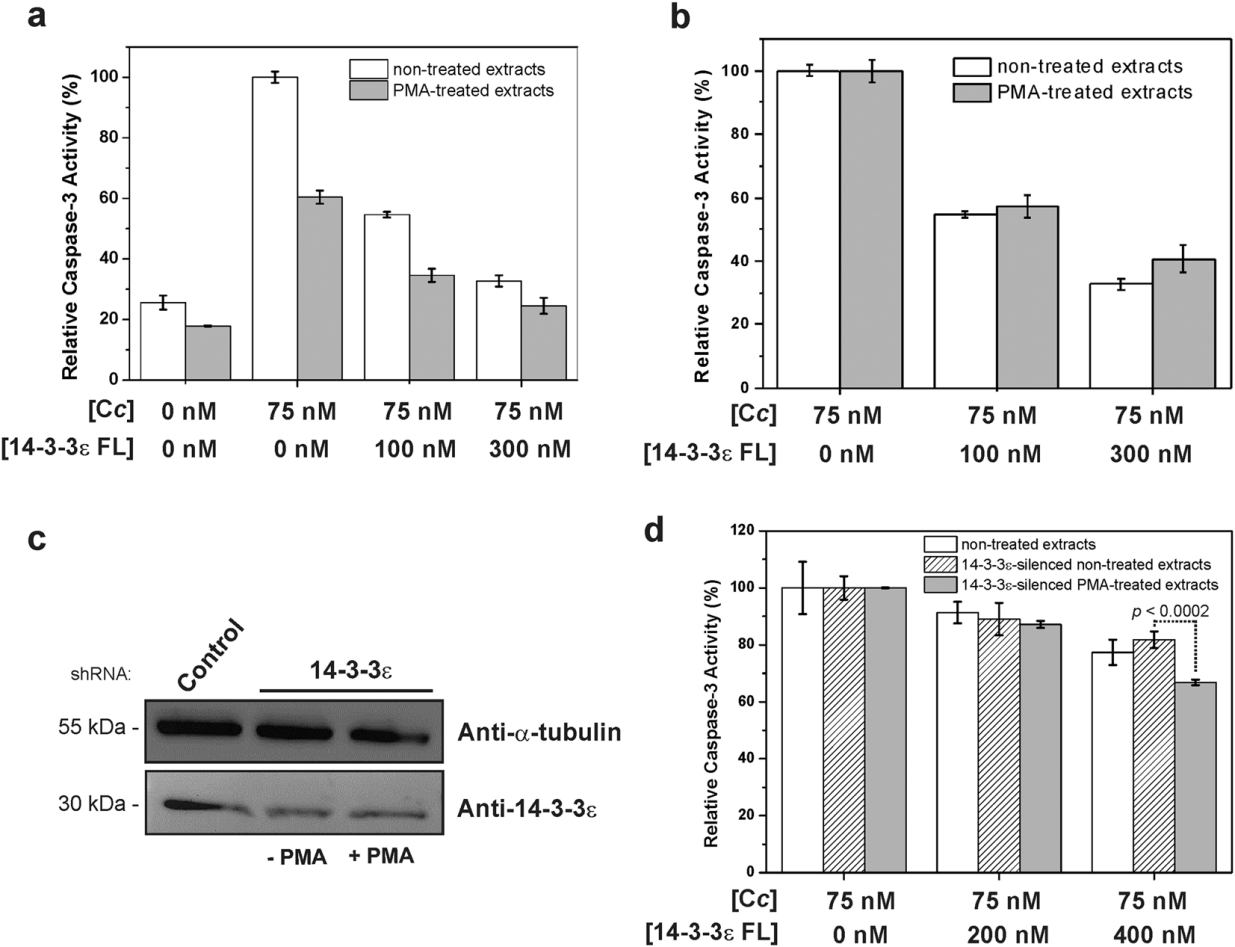
14-3-3ε FL inhibits caspase-3 activity in HEK293T cytosolic cell extracts. **a** Relative caspase-3 activity in non-treated (white columns) and PMA-treated (gray columns) HEK293T cytosolic cell extracts were measured upon addition of recombinant C*c* and increasing concentrations of 14-3-3ε FL. **b** Normalized data shown in **a**. **c** HEK293T cells were transfected with empty pSUPER (Control) or with pSUPER-shRNA against 14-3-3ε and immunoblotted with an anti-14-3-3ε antibody to examine the amount of remaining endogenous 14-3-3ε. An immunoblotting with anti-α-tubulin was used as loading control. **d** Normalized caspase-3 activity in 14-3-3ε-silenced non-treated and PMA-treated cell extracts upon addition of recombinant 14-3-3ε FL. Normalized caspase-3 activity of non-14-3-3ε-silenced, non-treated cell extracts is included as a control

First, we checked the ability of C*c* to activate caspase-3 in HEK293T cytosolic cell extracts. Caspase-3 activity was monitored upon C*c* addition (Fig. 2a, white columns), resulting in a substantial increase of such activity, as the hemeprotein triggered the apoptosome formation and, subsequently, caspase-9, -3 activation.

MAPK proteins modulate Apaf-1 activity, leading the activation of Rsk-1, which phosphorylates Apaf-1 at Ser268, favoring the interaction between Apaf-1 and 14-3-3ε, thereby increasing Apaf-1 inhibition^25^. Therefore, we tested the effect of phorbol 12-myristate 13-acetate (PMA), which triggers the MAPK cascade, on the caspase-3 activity. Apaf-1 phosphorylation under PMA treatment was confirmed by Phos-tag^TM^ SDS PAGE (Supplementary Figure S2). PMA-treated cells extracts showed a lower caspase-3 activity than untreated extracts in the presence of C*c* (Fig. 2a, gray columns). This is consistent with Apaf-1 phosphorylation at Ser268, enhancing its inhibition by 14-3-3ε^25^.

Then, we tested the ability of recombinant 14-3-3ε (herein named as 14-3-3ε full length, 14-3-3ε FL) to inhibit caspase-3 activity in non-treated and PMA-treated cell extracts. Addition of 14-3-3ε FL made extracts to exhibit significantly lower C*c-*mediated caspase-3 activity values (Fig. 2a). These were normalized to identify the particular effect of added 14-3-3ε FL but, unexpectedly, the normalization evinced that the inhibition exerted by 14-3-3ε FL was similar in non-treated and PMA-treated extracts (Fig. 2b). One of the plausible explanations for this observation is endogenous 14-3-3ε already abolished the caspase-3 activity by preferentially binding to phosphorylated Apaf-1, thus preventing any additional effect of exogenous 14-3-3ε FL. Additional caspase-3 assays were carried out using extracts from 14-3-3ε knockdown HEK293T cells (Fig. 2c), with the caspase-3 activity in PMA-treated extracts resulting more sensitive than non-treated extracts to the addition of 14-3-3ε FL (Fig. 2d).

In summary, these findings demonstrate how 14-3-3ε FL inhibits caspase activity, and how this inhibitory mechanism can be modulated by Apaf-1 phosphorylation. Nevertheless, the inhibitory effect of 14-3-3ε FL on caspase-3 could be caused by either a direct interaction with Apaf-1 or by hindering the complex between the latter and C*c*. Caspase-9 can indeed be phosphorylated by the ERK MAPK pathway, with the resulting modulation of its function and consequently, of caspase-3 activation^26^.

**Fig. 3:**
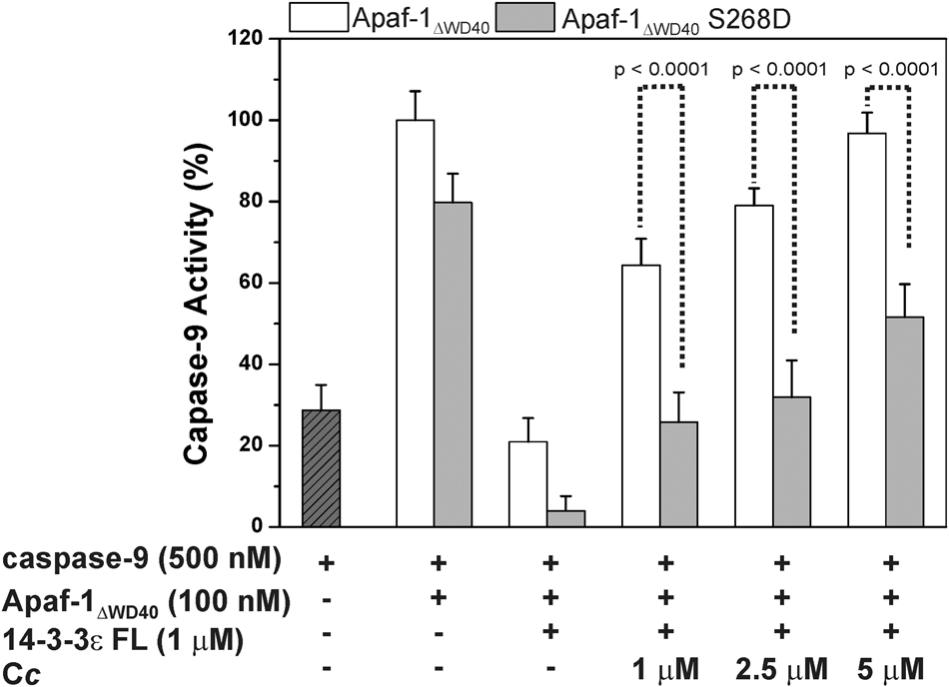
C*c* impairs 14-3-3ε FL-mediated caspase-9 inhibition. C*c* and 14-3-3ε FL were added to caspase-9 and different Apaf-1_ΔWD40_ mutants

To discard the direct interaction between C*c* and Apaf-1, as well as the ERK-mediated inhibition of caspase-9, we performed caspase-9 activity assays using a recombinant, truncated form of Apaf-1 lacking the C*c*-binding WD40 domains (Apaf-1_ΔWD40_)^27^. Apaf-1_ΔWD40_ activates caspase-9 in a constitutive manner in the absence of C*c* (Fig. 3). The inability of C*c* to alter the Apaf-1_ΔWD40_-mediated caspase-9 activity was likewise determined (Supplementary Figure S3). Then, the addition of 14-3-3ε FL inhibited caspase-9 activity strongly, due to the binding of Apaf-1_ΔWD40_ to 14-3-3ε FL (Fig. 3). However, the sequentially titration with C*c* restored caspase-9 activity, indicating a competition between C*c* and Apaf-1_ΔWD40_ for the binding site of 14-3-3ε FL, impairing Apaf-1 inhibition. Nuclear magnetic resonance (NMR) experiments corroborated such a competition. Specifically, the ε-methyl group signal of Met80 in reduced C*c* substantially broadened upon binding of the hemeprotein to 14-3-3ε (Supplementary Figure S3e). Further addition of Apaf-1_ΔWD40_ yielded partial recover of the signal intensity, indicating that Apaf-1_ΔWD40_ competes with C*c* for the 14-3-3ε FL binding site. The inability of Apaf-1_ΔWD40_ to bind C*c* was corroborated (Supplementary Figure S3f).

As phosphorylation of Apaf-1 at Ser268 enhances its interaction with 14-3-3ε FL, we tested the ability of C*c* to modulate the 14-3-3ε FL / Apaf-1_ΔWD40_ complex upon phosphorylation. To this end, we designed the recombinant Apaf-1_ΔWD40_ S268D mutant, which mimics Ser268 phosphorylation. As a control of unspecific effects caused by mutations at such position, the Apaf-1_ΔWD40_ S268A variant was also tested. Notably, the ability of phosphomimetic Apaf-1_ΔWD40_ S268D mutant to trigger caspase-9 activity was similar to that exhibited by Apaf-1_ΔWD40_. Nevertheless, the Apaf-1_ΔWD40_ S268D-mediated caspase-9 activity was slightly more sensitive to 14-3-3ε (Fig. 3, gray columns). Indeed, the recovery of enzymatic activity by C*c* was substantially lower than that measured using Apaf-1_ΔWD40_ (Fig. 3, gray columns). Such a finding indicates a larger affinity of the phosphorylated Apaf-1 species toward 14-3-3ε that displaces C*c*^25^. On the other hand, the caspase-9 activation in the presence of the S268A mutant behaved in a way similar to that in the presence of Apaf-1_ΔWD40_ (Supplementary Figure S3b). Hence, the negative charge at position 268 in Apaf-1 is responsible for the increase in affinity of Apaf-1 toward 14-3-3ε FL.

**Fig. 4:**
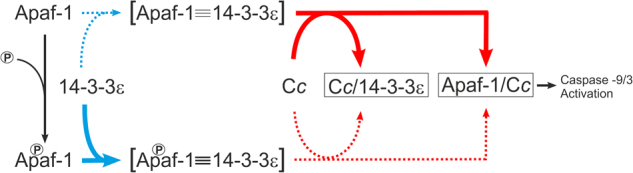
Model of Apaf-1 regulation by C*c* and 14-3-3ε. Model of Apaf-1 regulation by C*c* and 14-3-3ε. During homeostasis, Apaf-1 is available to interact with 14-3-3ε^25^. Upon its release into the cytosol, C*c* competes with Apaf-1 for the binding to 14-3-3ε, impairing the Apaf-1 / 14-3-3ε complex. Thus, Apaf-1 is available to interact with C*c*, thereby activating the caspases cascade^6^. Upon Apaf-1 phosphorylation, the equilibrium of the reaction is strongly shifted toward the Apaf-1 / 14-3-3ε complex formation. This would hinder the competition performed by C*c*, thereby partly impairing the activation of the caspases

Our results thus support a model in which C*c* activates Apaf-1 while blocking the inhibition exerted by 14-3-3ε on the apoptotic factor (Fig. 4).

### C*c* recognizes two different binding sites of 14-3-3ε full length

The dimerization state of 14-3-3ε FL was confirmed by analytic ultracentrifugation combined with dynamic light scattering (Supplementary Figure S4a-c). Sedimentation velocity experiments confirmed the dimeric state of 14-3-3ε FL with a molecular mass of 66.4 kDa (Supplementary Figure S4b). Dynamic light scattering measurements corroborated that 14-3-3ε FL behaves as a dimer, with a particle size of 9 ± 2 nm (Supplementary Figure S4c).

Isothermal titration calorimetry (ITC) experiments in Supplementary Figure S5 revealed that C*c* binds to 14-3-3ε FL homodimer with a 2:1 stoichiometry and a dissociation constant (*K*_D_) of 2.3 μM (Supplementary Table S1). To reveal 14-3-3ε residues implicated in the interaction with C*c*, 14-3-3ε FL D21K, K50E, S59E, E92K, D99K, and S187D mutants were designed. Residues were selected according to their role in the interactions of 14-3-3 isoforms with their respective partners^28–30^ and the impact of each mutation in the electrostatic surface potential of the protein. The dimeric state of these mutants was confirmed by analytic ultracentrifugation and dynamic light scattering (Supplementary Figure S4c and d). According to ITC analysis (Supplementary Table S1 and Figure S5), the six mutants bound to C*c* with slightly larger *K*_D_ values than 14-3-3ε FL WT. Further, the complexes involving 14-3-3ε FL D21K, K50E, S59E, or E92K showed a 1:1 stoichiometry instead of the 2:1 found for the WT species (Supplementary Table S1), suggesting that one of the two C*c*-binding sites of 14-3-3ε FL has been compromised in these mutants. Therefore, Asp21, Lys50, Ser59, and Glu92 residues may be essential in C*c* recognition.

**Fig. 5:**
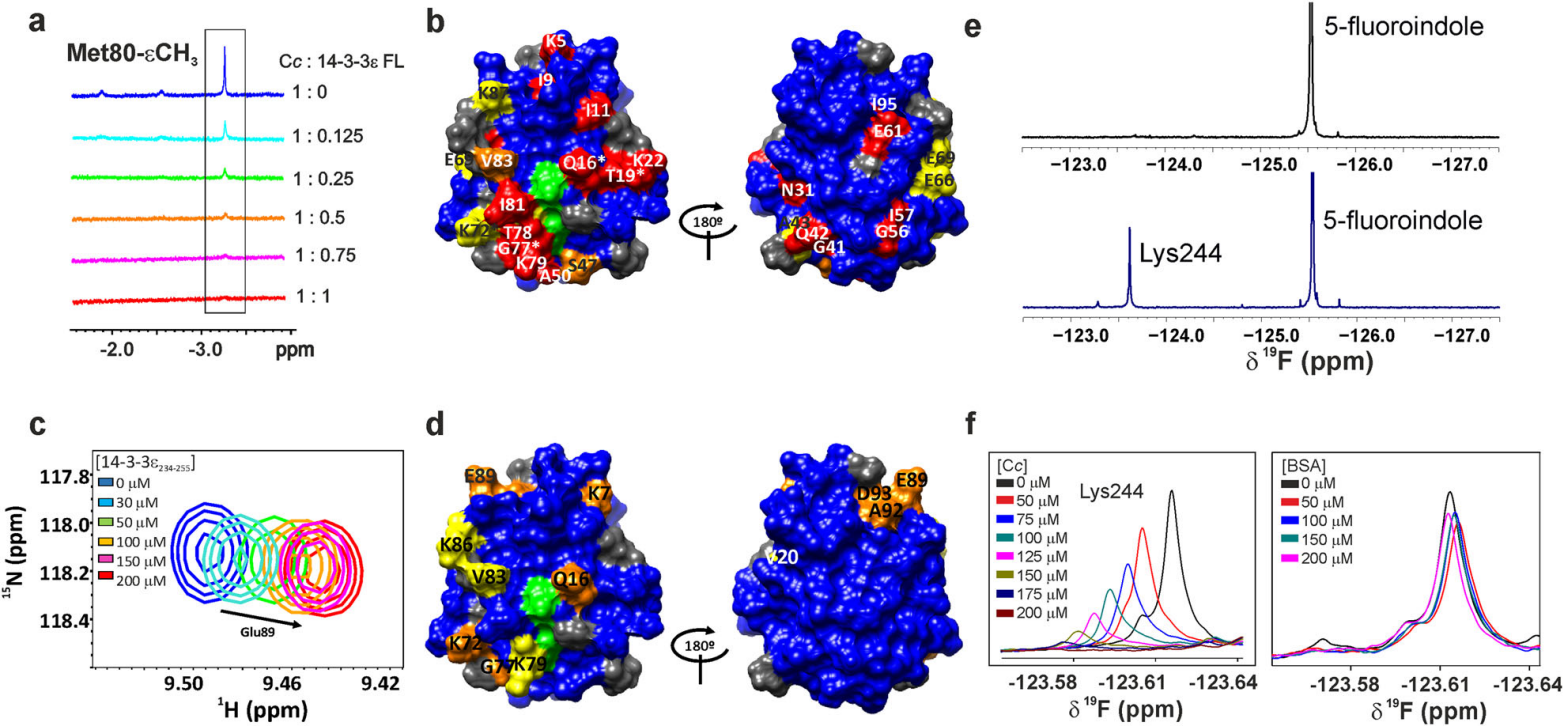
Binding of C*c* to 14-3-3ε as analyzed by NMR. **a** 1D ^1^H NMR spectra of C*c* (Fe^2+^) along the titration with 14-3-3ε FL. The complex formation is monitored by line broadening of the Met80-εCH_3_ signal, included in the black square. **b** Mapping of the C*c* residues perturbed upon binding to 14-3-3ε FL. The C*c*:14-3-3ε ratio is 1:0.75. Residues are colored according to their ^1^H ΔΔν_1/2Binding_ (Supplementary Figure S6b). Residues with average chemical-shift perturbations (Δδ_avg_) larger than 0.035 p.p.m. are represented in orange. Asterisks stand for residues showing both significant ^1^H ΔΔν_1/2Binding_ and Δδ_avg_. Residues without substantial ^1^H ΔΔν_1/2Binding_ and Δδ_avg_ are in blue. **c** Detail of superimposed 2D [^1^H, ^15^N] HSQC spectra of ^15^N-labeled C*c* along titration with 14-3-3ε_234-255_. Signals corresponding to distinct titration steps are colored according to the code in the panel. Arrow indicates direction of CSPs. **d** CSP map of reduced C*c* upon addition of 14-3-3ε_234-255_. Residues are colored according to Δδ_avg_ categories: insignificant Δδ_avg_ < 0.020 ppm., blue; small 0.020 ppm. ≤ Δδ_avg_ < 0.025 ppm. yellow_;_ and large Δδ_avg_ > 0.025 ppm., red. In **b**, **d** prolines and non-assigned residues are colored in gray; whereas, the heme group in green. **e** 1D ^19^F NMR spectra of free 5-fluorindole (upper) and when it is conjugated with 14-3-3ε_234-255_ peptide (lower). **f** 1D ^19^F NMR spectra of 14-3-3ε_234-255_ whose Lys244 is covalently bound to 5-fluorindole free and in the titrations with C*c* and BSA. ^19^F CSPs are colored according to the panels

We also performed NMR experiments to further characterize the interaction between reduced C*c* and 14-3-3ε FL. We followed C*c* signals upon titration of 14-3-3ε FL, which caused an overall broadening of C*c* resonances because C*c* / 14-3-3ε FL complex formation (Fig. 5a and Supplementary Figure S6a, left). Specifically, the ε-methyl group signal of the Met80 C*c* residue was largely broadened at 1:0.5 C*c*:14-3-3ε FL ratio (Fig. 5a), in agreement with the stoichiometry inferred from ITC data. Moreover, specific C*c* backbone amide groups underwent singularly large changes in their ^1^H line widths (^1^H ΔΔν_1/2 Binding_) upon binding with 14-3-3ε FL (Supplementary Figure S6b). These resonances were expected to be at or in the proximity of the area of C*c* involved in the recognition of 14-3-3ε FL. Moreover, several signals displayed substantial chemical-shift perturbations (CSPs) in the presence of 14-3-3ε FL (Supplementary Figure S6a, right). Specifically, Gln16, Thr19, Ser47, Lys72, Gly77 and Val83 signals showed significant CSPs (Δδ_avg_ > 0.035 ppm.). Interestingly, the signal corresponding to Val83 (Supplementary Figure S6a, right) seems to be in conformational exchange in the free C*c*, fixing its conformation upon the interaction with 14-3-3ε. These effects are consistent with an intermediate exchange rate at the NMR time scale, in agreement with the transient nature of this complex and the *K*_D_ values in the μM range.

Both broadening and CSPs maps on C*c* surface (Fig. 5b) indicate that C*c* mainly recognizes 14-3-3ε FL by a surface patch surrounding the heme cleft, as it does in its homeostatic interactions in the electron transport chain^31–33^.

### The C-terminal tails of 14-3-3ε are important in the interaction with C*c*

To decipher the role of the unstructured C-terminal tail of 14-3-3ε FL during C*c* recognition, the interaction between C*c* and the 14-3-3ε core protein, lacking C-terminal tails, was measured by ITC. The recorded thermograms showed very similar thermodynamic parameters for both 14-3-3ε FL and 14-3-3ε core proteins interacting with C*c*, although a slight increase in the *K*_D_ value was observed in the interaction between C*c* and the 14-3-3ε core (Supplementary Table S1). This suggests that the C-terminal tails of 14-3-3ε may participate in the interaction with C*c*, despite being unessential.

To confirm that the C-terminal tails of 14-3-3ε (named 14-3-3ε_234-255_) are involved in the interaction with C*c*, 2D [^1^H, ^15^N] HSQC were recorded along a titration of 14-3-3ε_234-255_ peptide onto ^15^N-labeled reduced C*c* (Supplementary Figure S6c). Such titration resulted in significant CSPs of the C*c* amide signals (Fig. 5c). The resulting interaction surface (Fig. 5d) indicates that 14-3-3ε_234-255_ binds to the rim of the heme groove of C*c*, as with 14-3-3ε FL protein. However, unlike the 14-3-3ε FL interaction, most of the C*c* resonances were in fast exchange on the NMR time scale upon binding to the 14-3-3ε_234-255_ peptide. ^1^H Δδ_bind_ was fitted using the best 1:1 binding model, obtaining a *K*_D_ of 49 μM (Supplementary Figure S6d). Moreover, ITC experiments in which the 14-3-3ε_234-255_ peptide was titrated onto C*c* yielded a *K*_D_ value of 16 μM (Supplementary Figure S5 and Table S1). Both NMR and ITC *K*_D_ values are marginally higher than the value obtained for the interaction between 14-3-3ε FL and C*c*. Furthermore, the ITC analysis also confirmed a 1:1 stoichiometry.

Then, to easily determine a residue which could be included in the latter docking calculations, the only one positively charged residue (Lys244) of 14-3-3ε_234-255_ peptide was tagged with 5-fluorindole molecule through a conjugation reaction. To prevent the reaction of 5-fluorindole with the N-terminal −NH_3_^+^ group, the peptide was modified by acetylation. To confirm that the acetylation in the N-terminal residue did not drastically modify the interaction with C*c*, ITC experiments were performed with the acetylated 14-3-3ε_234-255_. Both the non-acetylated and acetylated peptides interacted with C*c* in a similar way, despite the *K*_D_ value showed a slight increase when the peptide was acetylated (Supplementary Table S1).

Proper folding of 14-3-3ε_234-255_ before and after the conjugation reaction was unambiguously assessed by 1D ^1^H NMR spectra (Supplementary Figure S6e). Second, 1D ^19^F NMR spectra of both free 5-fluorindole and fluorinated 14-3-3ε_234-255_ were registered. The spectrum of fluorinated 14-3-3ε_234-255_ peptide showed a signal at −125.53 ppm. corresponding to free 5-fluorindole, and another at −123.62 ppm. that was assigned to 5-fluorindole conjugated to the epsilon −NH_3_^+^ group of Lys244 (Fig. 5e). The addition of increasing concentration of C*c* resulted in significant CSPs of this second signal (Fig. 5f, left panel). Notably, the ^19^F NMR signal also experienced a significant broadening consistent with the larger correlation time of the peptide bound to C*c*.

As a negative control, bovine serum albumin (BSA) protein was titrated into fluorinated 14-3-3ε_234-255_ peptide, with the negligible resulting ^19^F CSPs, suggesting that BSA cannot significantly bind to the peptide. (Fig. 5f, right panel). These results agree with Lys244 of 14-3-3ε FL taking part in C*c* recognition. Therefore, Lys244 was included as an active residue in further docking calculations (see below).

### Structural model of the interaction between C*c* and 14-3-3ε full length

**Fig. 6:**
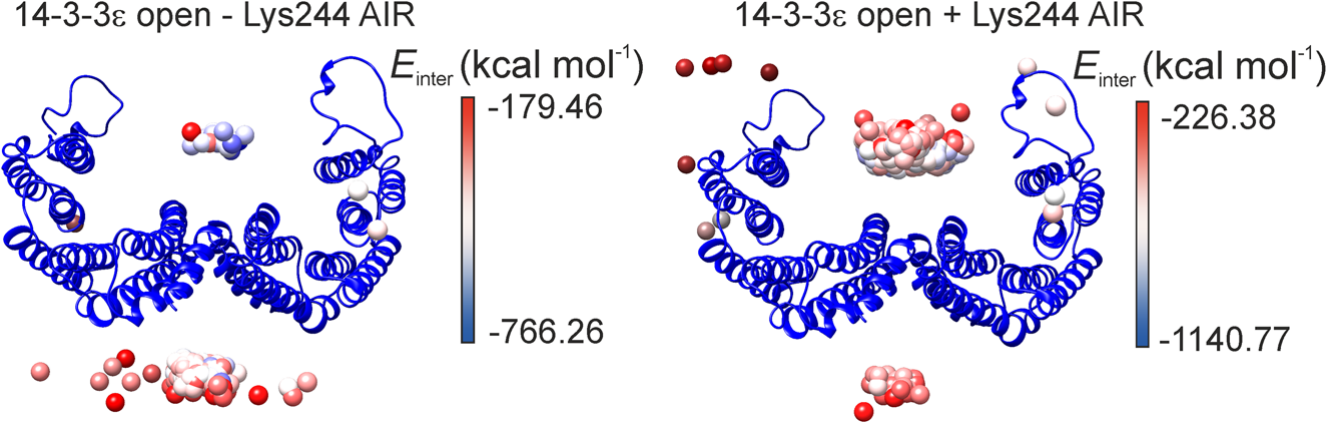
HADDOCK molecular docking of C*c* and 14-3-3ε FL. Mass centers of C*c* on the structural model of 14-3-3ε FL on its open conformation, including (right) or not (left) Lys244 as an active residue (AIR). Mass centers of C*c* are colored as a function of intermolecular energy (E_inter_) according to the scales represented in their corresponding panels

To further understand the molecular features of the interaction between C*c* and 14-3-3ε FL, we performed restrain-driven docking computations using HADDOCK software^34^. For these computations, we used the structural model of 14-3-3ε FL in its open conformation inferred from MD as the input structure (Supplementary Information and Figure S7), to allow the entrance of C*c* into the concave groove of the 14-3-3ε dimer. Line widths and CSPs obtained from NMR analysis of the C*c* / 14-3-3ε FL complex were used as C*c* ambiguous interaction restrains (AIRs). The 14-3-3ε FL residues that resulted in the impairment of one of the C*c*-binding sites by ITC were used as 14-3-3ε AIRs. The 500 solutions obtained in the computation showed two main C*c*-binding sites on 14-3-3ε FL dimer, in agreement with the stoichiometry inferred from ITC data. These solutions located C*c* in both the concave and convex faces of 14-3-3ε; these interacting regions will be referred hereafter to as the concave and convex binding sites, respectively. The convex site resulted more populated, although some of the lowest energy solutions locate within the concave site (Fig. 6, left panel). Statistical classification of solutions yielded three clusters in the convex site of 14-3-3ε and only one in the concave site (Supplementary Figure S8a). Further analyses indicate that electrostatics is the major contributing factor to binding energy in the three clusters (Supplementary Table S2). However, the concave cluster was more energetically favored than the convex clusters, mainly due to a lower electrostatic energy (E_elec_). Nevertheless, convex clusters presented lower AIRs violations than the concave cluster.

Furthermore, to study the role of the 14-3-3ε C-terminal tails in the interaction, a docking computation was also performed including Lys244 (located in the C-terminal tail), as an active residue. This computation showed an increase in the number of solutions locating C*c* in the concave binding site of 14-3-3ε (Fig. 6, right panel), suggesting that the C-terminal tails contribute to the entrance of C*c* into the concave groove of 14-3-3ε. Clustering analysis exhibited three clusters of solutions presenting C*c* bound to the concave site of 14-3-3ε, and only one displaying C*c* bound to the convex site (Supplementary Figure S8b). Again, electrostatic interactions constituted the major contribution in all clusters, concave site clusters displaying lower energies because of their E_elec_ term (Supplementary Table S2).

Structural models of C*c* / 14-3-3ε FL complex were obtained for computations excluding and including (Supplementary Figure S8c and d, respectively) Lys244 as an active residue of 14-3-3ε. For this, the best two complexes with minimal E_inter_ at each binding site of 14-3-3ε were selected from each computation. Both structural models present a C*c* molecule located in the concave groove of 14-3-3ε, contacting at least one of the C-terminal tails of 14-3-3ε dimer. A second C*c* molecule is placed at the convex side of 14-3-3ε, specifically at the rim of the dimerization surface thereby binding both 14-3-3 monomers. In addition, the two C*c* molecules from both computations present their heme clefts oriented to 14-3-3ε, according to NMR data.

## Discussion

Due to the apoptosis significance in multicellular organisms, its regulation relies on a subtle balance between inhibition and activation pathways. In fact, signaling cascades convert continuous stimuli in an all-or-none response to avert physiological fluctuations to trigger a response accidentally^35^. Feedbacks are needed to lead response ultra-sensitivity: a large stimulus is required to trigger a response, which changes dramatically within a narrow range of stimulus intensity. During homeostasis, apoptotic factors remain inhibited, either blocked by other proteins or sequestered in a particular cell compartment. Along apoptosis, several apoptotic factors are released from the mitochondria to the cytosol. Among these factors is C*c*, which promotes the apoptosome assembly through its association with Apaf-1, thereby initiating the caspase cascade^6^. On the other hand, pro-survival pathways, as the inhibition of Apaf-1 by 14-3-3ε^25^, reduce the sensitivity to the apoptosis signaling network.

Therefore, interactions between pro-apoptotic and pro-survival proteins, such as that between C*c* and 14-3-3ε, are of particular relevance as they provide additional regulation loops. In this study, we have shown that C*c* interacts with 14-3-3ε in the cytosol under apoptotic conditions, preventing the binding of 14-3-3ε to a specific site in Apaf-1. Hence, this interaction could be capable of regulating caspase activity. Indeed, C*c* enhanced caspase activation in presence of 14-3-3ε inhibitor and a truncated, constitutive form of Apaf-1 unable to bind the hemeprotein. Thus, C*c* plays a double role in the activation of caspase cascade directly by interacting with Apaf-1, and indirectly by blocking 14-3-3ε thereby releasing Apaf-1. This double role enables C*c* to inhibit pro-survival pathways while apoptotic routes have been triggered (Fig. 4).

Further phenomena modulate the cell fate signaling, including protein phosphorylation events, as those which regulate C*c* functions, including its ability to activate caspases^36–40^. In addition, such a phosphorylation enhances the inhibition of Apaf-1 by 14-3-3ε^25^, making caspase activation less sensitive to C*c*. In fact, activation of MAPK cascade favors the Apaf-1 inhibition by 14-3-3ε. Similarly, the phosphomimetic Apaf-1 mutant is more susceptible to 14-3-3ε-dependent inhibition, which consequently becomes less sensitive to the presence of C*c*. Hence, this phosphorylation of Ser268 in Apaf-1 may attenuate the effect of C*c* release when caspase activation is undesirable (Fig. 4).

C*c* does lack the consensus sequence which 14-3-3ε usually recognizes in its various targets. Our biophysical analysis has revealed that two molecules of C*c* can interact with 14-3-3ε at two different sites: the groove of 14-3-3ε where its targets bind, and the convex side of the dimer. Additionally, the data herein suggest that flexible C-terminal 14-3-3ε may recognize C*c* without being essential for the binding. These tails are flexible and mostly unstructured, although MD trajectories indicate the presence of residual helical elements. Moreover, NMR data show that C*c* recognizes 14-3-3ε by residues surrounding the heme cleft, as reported for other interactions involving the hemeprotein^31–33^.

In summary, C*c* is revealed as a double Apaf-1 activator via two mechanisms: by directly binding to the apoptosis factor and impairing its inhibition by 14-3-3ε. Indeed, such a cooperative behavior of C*c*, triggering effector pathways while modulating feedbacks loops make cell response ultrasensitive to its release. The analysis of the various additional interactions of C*c* during the onset of programmed cell death recently unveiled may confirm if this conclusion can be extended as a general mechanism by which C*c* triggers apoptosis^9–12,41^.

## Materials and methods

### Cell cultures

HeLa and Human Embryonic Kidney 293T (HEK293T) cells were cultured in Dulbecco’s modified Eagle’s medium (DMEM), supplemented with 10% heat-inactivated fetal bovine serum (FBS), 2 mM l-glutamine, 100 U/mL penicillin, 100 μg/mL streptomycin, and maintained at 37 °C in a humidified 5% CO_2_ atmosphere.

For caspase-3 assays, 4.5 × 10^6^ HEK293T cells were cultured in 140 mm Petri dishes in DMEM containing FBS until 70% confluence was reached, and cells were then kept in DMEM without FBS for 4 h. In some experiments (indicated in the main text) cells were treated with 50 ng/mL phorbol-12-myristate-13-acetate (PMA, Sigma-Aldrich) or treated with the same volume of DMSO for 120 min.

### shRNA knockdown

In order to knockdown 14-3-3ε expression in HEK293T cells, a construct containing the shRNA sequence (5′-GATCCCCTGTTCAATACTGCTGATTATTCTCCTTCAAGAGAGGAGAATAATCAGCAGTATTGAACATTTTTA-3′) was cloned into a pSUPER vector using *Bg*III–*Hind*III restriction sites. An empty pSUPER vector was used as a control. Cells were transfected with the corresponding pSUPER plasmid by calcium phosphate method, as described elsewhere^42^. Transfected cells were selected by adding puromycin (1.5 μg/mL) to the culture medium. Four days after transfection, cells were collected and cytosolic extracts were obtained.

### Subcellular fractionation and extract preparation

For immunoprecipitation assays, 6 × 10^6^ HeLa cells were seeded in 140 mm Petri dishes and treated with an apoptosis inducing agent (20 μM camptothecin, CPT) for 4 h. HeLa cells were fractionated into cytosolic, membrane/organelles and nuclear fractions using a ProteoExtract Subcellular Proteome Extraction Kit (Calbiochem) following the manufacturer’s indications. The purity of subcellular fractions obtained was verified by western blot analysis, using anti-α-tubulin and anti-COX IV for detecting cytosol and mitochondria, respectively.

For caspase-3 activity assays, cytosolic extracts were obtained from HEK293T cells as previously described with briefly modifications^43^. Briefly, cells were trypsinized and collected by centrifugation (5 min at 1500×*g*) and washed twice with phosphate buffer saline (PBS) and once with Cellular Extract Buffer (CEB, 20 mM HEPES at pH 7.5, 10 mM KCl, 1.5 mM MgCl_2_, 1 mM Na-EDTA, 1 mM EGTA, 1 mM dithiothreitol [DTT], 100 μM PMSF). Two volumes of CEB were added to cells, and they were incubated for 15 min on ice, allowing the hypotonic swell of cells. The cells were then disrupted by 20–25 strokes with a Douncer homogenizer and lysates were centrifuged at 15,000×*g* for 15 min at 4 °C to remove organelles.

### Immunoprecipitation

Cytosolic fractions from subcellular fractionation of control and CPT-treated HeLa cells were used to immunoprecipitate C*c*. Quantity of 300 μg of cytosolic samples were incubated with 50 μL Sepharose 6B (Sigma-Aldrich) for 2 h at 4 °C under agitation as a pre-clearing step to reduce non-specific binding. Samples were centrifuged and 20 μL of rabbit anti-human cytochrome *c* (C*c*) were added to supernatants and incubated overnight at 4 °C under rotation. As a negative control, 300 μg of cytosolic lysates were incubated with 1 μg of anti-mouse IgG under the same conditions. Afterwards, 50 μL Protein A Sepharose (GE Healthcare) was added to each lysate for 4 h at 4 °C under rotation. The protein-Sepharose complexes were washed extensively, collected by centrifugation and boiled in freshly prepared reducing loading buffer. Controls including 30 μg of cytosolic lysates were run concurrently with the immunoprecipitated (IP) samples in the western blot assays.

Apaf-1 immunoprecipitation was performed from total non-treated and PMA-treated lysates. Apaf-1 IP samples were analyzed by western blot using a Phos-tag^TM^ SDS PAGE (Wako) — which reduce the electrophoretic mobility of phosphorylated proteins.

### Antibodies

Rabbit anti-human C*c* serum was obtained after immunizing male rabbits with full length recombinant C*c* suspended in a 0.85% solution of NaCl (20 mg/mL). The suspension was then incorporated into an equal amount of complete (Freund) adjuvant obtained from Difco (BD Biosciences, US). Mouse monoclonal anti-α-Tubulin (catalogue number T8328), as well as secondary horseradish peroxidase (HRP)-conjugated anti-mouse IgG (catalogue number A4416), rabbit polyclonal anti-14-3-3ε (catalogue number SAB4503100), and anti-rabbit IgG (catalogue number A0545) were obtained from Sigma-Aldrich. Rabbit polyclonal antibody to cytochrome *c* oxidase subunit IV (COX IV) was from Abcam (catalogue number ab16056). Mouse monoclonal anti-Apaf-1 (catalogue number sc-135836) was obtained from Santa Cruz Biotechnology, INC. Mouse monoclonal anti-phosphoserine (Millipore clone A4) was obtained from Merck.

### Western blot analysis

For the immunoblot detection of C*c*, α-Tubulin, COX IV, and 14-3-3ε in the subcellular fractions or IP samples, protein content was measured using the DC^TM^ protein assay (Bio-Rad Laboratories, US). Proteins were resolved by sodium dodecyl sulphate-polyacrylamide gel electrophoresis (SDS-PAGE) in 12% gels and then transferred onto polyvinylidene fluoride (PVDF) membranes (EMD Millipore) using a Mini Trans-Blot electrophoretic transfer cell (Bio-Rad). Membranes were blocked in 5% non-fat dry milk in PBS with Tween-20 (TPBS) and immunoblotting was performed with primary antibodies. HRP-conjugated secondary antibodies were used for detection. The immunoreactive bands were detected using Amersham ECL Plus Western Blotting Detection Reagents (GE Healthcare Life Sciences).

### Cloning, expression, and purification of recombinant proteins

C*c* was expressed in *Escherichia coli* BL21 (DE3) strain as previously described^32^. First, 25 mL Luria-Bertani (LB) medium, supplemented with 100 μg/mL ampicillin, were grown overnight at 37 °C under agitation. Volume of 2.5 mL of pre-culture was used to inoculate 2.5 L of LB medium. The culture was incubated at 30 °C under agitation for 24 h. Then, cells were centrifuged at 6000 r.p.m. for 10 min in an Avanti J-25 centrifuge (Beckman Coulter). Following this, cells were re-suspended in 1.5 mM borate buffer (pH 8.5), sonicated for 4 min and, finally, cellular debris was eliminated by centrifugation (20,000 r.p.m. for 30 min). For NMR measurements, ^15^N-labeled C*c* was produced in minimal media with ^15^NH_4_Cl as a unique nitrogen source. C*c* purification was carried out by ionic chromatography with a carboxy-methylcellulose matrix. Fractions containing C*c* were concentrated in an Amicon (3 kDa cutoff) until correct concentration was achieved.

14-3-3ε full length (14-3-3ε FL) was cloned into the pET-28a vector under the T7 promotor using *Nde*I – *Xho*I restriction sites and containing a N-terminal hexahistidine tag. For cloning, the following primers were used: 5′-AGCCATATGATGGATGATCGAGAGGAT-3′ and 5′-GTGCTCGAGTCATTCCTGATTTTCGTC-3′. The 14-3-3ε core was cloned with a Stop codon introduced after residue at position 233. The primers used for this mutagenesis were 5′-ACACTATGGACTTCATAGATGCAGGGTGACGGT-3′ and 5′-ACCGTCACCCTGCATCTATGAAGTCCATAGTGT-3′. 14-3-3ε FL D21K, K50E, S59E, E92K, D99K, and S187D mutants were obtained from Mutagenex Inc. (USA). The expression of 14-3-3ε species were performed in the *E. coli* BL21 (DE3) strain. Cultures were grown in LB medium at 37 °C until an O.D._600 nm_ of 0.6–0.8 was reached. Protein expression was induced by adding 1 mM isopropyl-β-D-1-thiogalactopyranoside (IPTG) and cultures were grown for 24 h at 30 °C. The cells were collected by centrifugation and re-suspended in 20 mM Tris-HCl (pH 8), 800 mM NaCl, 10 mM imidazole. Cells were then broken by sonication and insoluble debris was removed by centrifugation, as indicated above. 14-3-3ε species were purified by affinity chromatography, using a Ni Sepharose 6 Fast Flow (GE Healthcare) column and an imidazole gradient ranging from 10 mM to 300 mM imidazole. Protein containing fractions were concentrated in an Amicon (3 kDa cutoff) until the desired proper protein concentration was reached.

Proteins were dialyzed against 10 mM sodium phosphate (pH 7.4) buffer for ITC measurements or 5 mM sodium phosphate (pH 6.3) buffer for NMR titrations. For caspase assays, protein was dialyzed against 20 mM HEPES (pH 7.5) buffer.

14-3-3ε C-terminal peptide (14-3-3ε_234-255_) was purchased from Genosphere Biotechnologies (France).

Apaf-1_ΔWD40_ S268D and S268A mutants were obtained by mutagenic PCR from pET28a-Apaf-1_ΔWD40_, using 5′-ACCAGAGACAAGGATGTTACAGATT-3′ and 5′-AATCTGTAACATCCTTGTCTCTGGT-3′ primers for the S268D mutant and 5′-ACCAGAGACAAGGCTGTTACAGATT-3′ and 5′-AATCTGTAACAGCCTTGTCTCTGGT-3′ primers for the S268A mutant. All Apaf-1_ΔWD40_ species were expressed as previously described with small modifications^6^. Protein expression was carried out in *E. coli* BL21 (DE3) at 20 °C after induction with 1 mM IPTG. Cells were broken by freeze/heat cycles and cellular debris was removed by centrifugation. The soluble fraction was purified by affinity chromatography (His·Trap column, GE Healthcare) followed by anionic-exchange (Hi·Trap column, GE Healthcare). Recombinant caspase-9 was expressed in *E. coli* BL21 (DE3) at 30 °C and purified as indicated above for Apaf-1_ΔWD40_. Protein quantification was assessed using the Bradford protein assay^44^.

### Caspase-9 assays

Caspase-9 activity was measured as previously described^6^. Recombinant Apaf-1_ΔWD40_ species and 14-3-3ε FL were mixed in buffer (40 mM HEPES, pH 7.5, 20 mM KCl, 1 mM DTT) to a final concentration of 100 nM and 1 μM, respectively. C*c* was added at different concentrations (ranging from 1 to 5 μM). After incubation for 15 min at room temperature, recombinant caspase-9 was added to a final volume of 100 μL and final concentration of 500 nM. After incubation for another 15 min at 37 °C, the caspase-9 substrate LEHD peptide coupled to 7-amino-4-trifluoromethylcoumarin (Ac-LEHD-AFC, TOCRIS) was added at a final concentration of 50 μM. The increase in fluorescence resulting from Ac-LEHD-AFC cleavage was determined in a Cary Eclipse (Varian) fluorescence spectrophotometer, using an excitation wavelength of 390 nm and an emission wavelength of 510 nm. Each experimental data were the mean ± SD value of at least three independent measurements.

### Caspase-3 assays

In vitro caspase-3 assays were performed as formerly described with slight changes^45^. Recombinant C*c* and 14-3-3ε FL were mixed and incubated with 100 μg of cytosolic HEK293T cells extracts for 60 min at 37 °C in 10 mM HEPES (pH 7.5) buffer, supplemented with 25 mM KCl, 200 μM DTT, 200 μM dATP. After incubation, 10 mM HEPES (pH 7.5), 50 mM NaCl, 40 mM β-glycerophosphate, 2 mM MgCl_2_, 5 mM EGTA, 0.1 mg mL^−1^ bovine serum albumin, and 0.1% (w/v) 3-[3-cholamidopropyldimethylammonio]-1-propanesulfonate (CHAPS) buffer was added to a final volume of 200 μL, supplemented with 40 μM of acetyl-DEVD-7-amino-4-methylcoumarin (Ac-DEVD-AMD, Enzo Life Sciences), a fluorescent substrate specific to caspase-3/7. The increase in fluorescence resulting from Ac-DEVD-AMC cleavage was determined in a Cary Eclipse (Varian) fluorescence spectrophotometer, using an excitation wavelength of 360 nm and an emission wavelength of 460 nm. To compare the effect of 14-3-3ε in different types of extracts, values for caspase-3 activity before adding recombinant 14-3-3ε FL were normalized. Each experimental data were the mean ± SD value of at least three independent measurements.

### ITC measurements

All ITC experiments were performed using an Auto-ITC200 (MicroCal-Malvern Instruments, UK) at 25 °C by titrating 14-3-3ε species and 14-3-3ε peptides with C*c*. The reference cell was filled with distilled water. The experiments consisted of 2-μL injections of 300 μM C*c* solution in 10 mM sodium phosphate buffer (pH 7.4) into the sample cell, initially containing 20 μM 14-3-3ε solution in the same buffer. All solutions were degassed before titrations were performed. Titrant was injected at appropriate time intervals to ensure the thermal power signal returned to the base line prior to the next injection. To achieve homogeneous mixing in the cell, the stirring speed was maintained constant at 750 r.p.m. The data, specifically the heat per injection normalized per mole of injectant vs. the molar ratio, were analyzed with Origin 7.0 (OriginLab Corp.). Calibration and performance tests of the calorimeter were carried out conducting CaCl_2_-EDTA titrations with solutions provided by the manufacturer. Control experiments (C*c* solution injected into buffer) were performed in order to assess potential unspecific heat effects.

### NMR spectroscopy

NMR titration of reduced C*c* with 14-3-3ε FL was followed by 1D ^1^H and 2D [^1^H, ^15^N] HSQC spectra on a Bruker Avance 700 MHz at 25 °C. 50 μM of reduced, ^15^N-labelled C*c* in 5 mM sodium phosphate buffer (pH 6.3) was titrated with unlabeled 14-3-3ε in the same buffer. 0.1 M sodium ascorbate and 10% D_2_O were added in order to ensure the redox state remained constant and to adjust the lock signal, respectively. Water signal was suppressed according to the WATERGATE solvent suppression method^46^. The interaction of reduced C*c* with 14-3-3ε_234-255_ was performed on a Bruker Avance 600 MHz in the same way as previously described for the titration. Data were processed using TopSpin NMR 2.0 software (Bruker). Line broadening and chemical-shift perturbation analysis were performed using Sparky 3 NMR Assignment Program (T.D. Goddard and D.G. Kneller, University of California – San Francisco, US). CSP titration curves were fitted using the one-site binding model.

To fluorinate the Lys244 side chain of 14-3-3ε_234-255_, the bioconjugation reaction was performed with 5-fluoroindole compound (Sigma) as previously described^47^. 500 μM acetylated 14-3-3ε_234-255_ peptide was mixed with 2 mM formaldehyde solution (37% w/w) and with 6 mM 5-fluoroindole for 24 h at 30 °C. Then, the sample was dialyzed against 10 mM sodium phosphate (pH 6.3) buffer overnight. Proper folding of 14-3-3ε_234-255_ before and after the reaction was checked by 1D ^1^H spectra, and the interaction with C*c* was followed recording 1D ^19^F experiments at 25 °C on a Bruker Avance 600 MHz.

### Analytical ultracentrifugation

Sedimentation velocity experiments of 14-3-3ε FL WT, D21K, K50E, S59E, E92K, D99K, and S187D were performed at 20 °C in an Optima XL-A Analytical Ultracentrifuge (AU, Beckman Instruments) with an AN50-Ti rotor. These experiments were carried out at 45,000 r.p.m. with 400-μL samples at 37 μM in 5 mM sodium phosphate buffer (pH 6.3) loaded into double sector cells. Radial scans at 280 nm were taken every 10 min and the sedimentation coefficient distribution was calculated by least squares boundary modeling of the sedimentation velocity data using the programme SEDFIT. The experimental coefficients were converted to standard conditions (s20,w).

### Molecular dynamic computations

Molecular dynamics (MD) computations were carried out using the AMBER 12 package and the AMBER-2003 force field. For the 14-3-3ε_234-255_ peptide simulation, an unfolded and outspread conformation was used as initial coordinates. After 100 ns of simulation, the closest structure to the average from the last 40 ns of this computation was attached to the crystallographic structure of 14-3-3ε (PDB: 2BR9)^15^ at the C-terminal of both monomers. The resulting structure was used as the initial coordinate for the full length protein in the MD computation. With the aim to promote the opening of 14-3-3ε FL and to produce an open conformation, both consensus peptide ligands for 14-3-3 proteins included in the crystallographic structure were removed from the initial coordinates.

All simulations were carried out under periodic boundary conditions using an orthorhombic cell geometry (minimum distance between protein and cell faces was initially set to 10 Å) and PME electrostatics were set with the Ewald summation cutoff at 9 Å. Sodium counter-ions were added to neutralize the charges of the system. The structures were solvated with SPC water molecules. Afterwards, solvent and counter-ions were subjected to 2000 steps of energy minimization followed by 300 ps NPT-MD computations using isotropic molecule position scaling and a pressure relaxation time of 1 ps at 298 K. The density of the system reached a plateau during the first 150 ps. Then, the whole system was energy minimized and submitted to NVT MD computations at 298 K. The SHAKE algorithm^48^ was used to constrain bonds involving hydrogen atoms. The PTRAJ module of AMBER was used for trajectory analysis.

### NMR-driven docking

Restrained docking calculations were performed with the High Ambiguity Driven Docking approach (HADDOCK)^49–51^, using the 3ZCF.pdb structure of C*c* and the resulting open model of 14-3-3ε FL from MD calculations. Dielectric constant was set as distance-dependent. Scaling of ambiguous interaction restraints (AIRs) were fixed to 0.1 for rigid body and to 1.0 for both semi-flexible simulated annealing and final refinement. AIRs for the docking simulation were generated using standard criteria. Random exclusion of AIRs was not employed. C*c* residues labeled as active were those showing ^1^H ΔΔν_1/2Binding_ ≥ 59.2 Hz and a solvent accessibility larger than 50% calculated with NACCESS. These active residues were Gln16, Gln42, Ser47, Ala50, Gly77, Lys79, Ile81, and Val83. C*c* residues located at <4 Å from the active ones and showing high-solvent accessible surface (>50%) were labeled as passive residues. 14-3-3ε residues labeled as active were those whose mutation resulted in the impairment of one of the C*c*-binding sites: Asp21, Lys50, Ser59, and Glu92. 14-3-3ε residues located at <4 Å from the active ones and showing high-solvent accessible surface (>50%) were labeled as passive residues. For each run, 10,000 rigid-body solutions were generated by energy minimization. The 1000 structures with the lowest AIRs energies were subjected to semi-flexible simulated annealing. The first 500 structures were submitted to a final refinement in explicit water. The 500 best structures were analyzed using standard criteria. AIR violations were calculated using as reference the solution with best HADDOCK score. Structures were represented using UCSF Chimera^52^.

### Dynamic light scattering measurements

Dynamic light scattering experiments were performed to evaluate the dimerization state of 14-3-3ε FL WT and D21K, K50E, S59E, E92K, D99K, and S187D mutants, in 5 mM sodium phosphate at pH 6.3. DLS experiments on 0.1 mg/mL of each 14-3-3ε species were conducted at 25 °C in a Zetasizer Nano ZS (Malvern Instruments).

### Statistical analysis

Statistical analysis was performed using GraphPad Prism 7 Software and experimental data of at least three independent measurements. Statistical significance was estimated using Student's *t*-test. A *p-*value of <0.0002 was considered significant.

## Electronic supplementary material

Supplementary Information
