# Supplementary material for: Cytochrome c speeds up caspase cascade activation by blocking 14-3-3ε-dependent Apaf-1 inhibition
Source: Cell Death Dis. 2018 Mar 6;9(3):365. doi: 10.1038/s41419-018-0408-1 (PMC5840378; doi:10.1038/s41419-018-0408-1)
Supplement: Supplementary file 1 — Supplementary Information [file 41419_2018_408_MOESM1_ESM.pdf]

Cytochrome *c* speeds up caspase cascade activation by blocking 14-3-3ε-dependent Apaf-1 inhibition

Supplementary Information

Carlos A. Elena-Real[1], Antonio Díaz-Quintana[1], Katiuska González-Arzola[1], Adrián Velázquez-Campoy[2], Mar Orzáez[3], Abelardo López-Rivas[4], Sergio Gil-Caballero[1], Miguel A. De la Rosa[1,*] and Irene Díaz-Moreno[1,*]

[1]Instituto de Investigaciones Químicas (IIQ) – Centro de Investigaciones Científicas Isla de la Cartuja (cicCartuja), Universidad de Sevilla – Consejo Superior de Investigaciones Científicas (CSIC), Sevilla, Spain.

[2]Institute of Biocomputation and Physics of Complex Systems (BIFI), Joint Unit IQFR-CSIC-BIFI, Universidad de Zaragoza, Zaragoza, Spain.

[3]Centro de Investigación Príncipe Felipe, Valencia, Spain.

[4]Centro Andaluz de Biología Molecular y Medicina Regenerativa-CABIMER, CSIC-Universidad de Sevilla-Universidad Pablo de Olavide, Sevilla, Spain.

***Corresponding authors:**

Emails: idiazmoreno@us.es; marosa@us.es

Telephone number: +34 954489513; Fax number: +34 954460065

Structural model of the 14-3-3ε *full length* protein in its open conformation

Several molecular structures of 14-3-3ε are available in the Protein Data Bank. All these structures show a homodimer that lacks the C-terminal tails corresponding to residues from 234 to 255. However, given the role of these tails in the recognition of C*c*, we built a structural model of 14-3-3ε FL, including the C-terminal tails, based on computational analysis.

First, we performed molecular dynamics (MD) calculations of the isolated C-terminal tail of 14-3-3ε (14-3-3$\varepsilon_{234-255}$). The root-mean-square deviation (RMSD) and radius of gyration (R_{gyr}) data remained stable after the first 20 ns (Supplementary Figure S7a). To obtain a representative structure of the 14-3-3$\varepsilon_{234-255}$ peptide during time with stable RMSD and R_{gyr} values, the structure with the lower RMSD respect to the average from the last 40 ns was selected (Supplementary Figure S7b). Despite being highly dynamic, the 14-3-3$\varepsilon_{234-255}$ peptide formed secondary structure elements, such as α-helices that remain throughout the computation.

The model of 14-3-3$\varepsilon_{234-255}$ peptide was bound to the crystallographic structure of 14-3-3ε_{1-233} (Supplementary Figure S1) to obtain an initial 14-3-3ε FL (stretch from 1 to 255 residues) model. Conformational changes have been previously described for 14-3-3 proteins enabling them to switch from closed to open conformations upon binding with their partners.[15] To obtain an open conformation that could accommodate C*c* (see below), MD calculations were carried out removing the two consensus ligands bound to 14-3-3ε present in the crystallographic structure (PDB: 2BR9). This guarantees flexibility and the opening of the protein structure.

The statistical parameters of 14-3-3ε FL showed high dynamism (Supplementary Figure S5c, *upper*). The large RMSD and R_{gyr} values (red lines) suggest that the behaviour of 14-3-3ε FL is considerably dynamic in this open state (Supplementary Figure S7c). On the contrary, the core of 14-3-3ε, ranging from residues 1 to 233, was characterized by RMSD values substantially smaller than those for 14-3-3ε FL, indicating that the C-terminal tails are highly dynamic (Supplementary Figure S7c, blue line).

The closest structure to the average was selected from the last 5 ns of MD computation (Supplementary Figure S7d). This resulting model of 14-3-3ε FL was used as input in the docking calculations.

SUPPLEMENTARY TABLES

Table S1: Thermodynamic values inferred from ITC measurements

**Protein complex**	K_D **(μM)**	**Δ*H* (kcal mol^{-1})**	***n***
**C*c* / 14-3-3ε FL WT**	2.3	1.4	1.70
**C*c* / 14-3-3ε FL D21K**	13.0	8.3	1.06*
**C*c* / 14-3-3ε FL K50E**	11.0	3.1	0.86*
**C*c* / 14-3-3ε FL S59E**	16.0	5.8	0.86*
**C*c* / 14-3-3ε FL E92K**	11.0	6.7	1.20*
**C*c* / 14-3-3ε FL D99K**	16.0	7.7	1.64
**C*c* / 14-3-3ε FL S187D**	12.0	1.3	1.50
**C*c* / 14-3-3ε core**	7.6	0.8	1.40
**C*c* / 14-3-3**$\varepsilon_{234-255}$	16.0	1.4	0.95
**C*c* / 14-3-3**$\varepsilon_{234-255(acetylated)}$	23.0	1.5	0.95

Thermodynamic equilibrium parameters for the interaction of *wild-type* and mutant 14-3-3ε species with reduced C*c*. Equilibrium dissociation constant (K_D), enthalpy (Δ*H*) and reaction stoichiometry (*n*) are shown. Asterisks indicate those stoichiometry values markedly lower than that for the interaction between C*c* and 14-3-3ε FL WT. Relative errors: K_D 20%, Δ*H* 5%.

Table S2. Statistical analysis of HADDOCK data after clustering the solutions

14-3-3ε open conformation - Lys244 AIR

Clusters	RMSD (Å)	Size	E_{inter} (kcal mol^{-1})	E_{vdw} (kcal mol^{-1})	E_{elec} (kcal mol^{-1})	AIRviol (Å)	BSA (Å^2)	Binding site on 14-3-3ε
**# 1**	1.96 ± 1.27	54	-309.41 ± 59.8	-35.15 ± 9.79	-349.94 ± 52.06	2.44 ± 1.15	1766 ± 233	Convex
**# 2**	4.98 ± 1.74	46	-290.34 ± 57.38	-34.09 ± 10.41	-351.16 ± 55.61	2.85 ± 0.95	1710 ± 264	Convex
**# 3**	2.13 ± 1.76	21	-281.97 ± 51.16	-29.05 ± 7.1	-312.93 ± 51.84	2.10 ± 0.87	1618 ± 190	Convex
**# 4**	6.34 ± 2.84	7	-385.26 ± 118	-21.30 ± 14.38	-556.57 ± 98.07	3.86 ± 1.55	1620 ± 396	Concave

14-3-3ε open conformation + Lys244 AIR

Clusters	RMSD (Å)	Size	E_{inter} (kcal mol^{-1})	E_{vdw} (kcal mol^{-1})	E_{elec} (kcal mol^{-1})	AIRviol (Å)	BSA (Å^2)	Binding site on 14-3-3ε
**# 1**	4.20 ± 1.66	35	-345.88 ± 90.5	-32.17 ± 10.12	-454.14 ± 87.43	3.57 ± 1.52	1709 ± 235	Concave
**# 2**	1.77 ± 0.69	30	-252.51 ± 66.72	-23.20 ± 6.09	-451.22 ± 67.87	5.07 ± 1.29	1380 ± 113	Concave
**# 3**	3.45 ± 1.18	24	-327.26 ±77.21	-24.00 ± 10.21	-460.74 ± 77.34	3.54 ± 1.00	1572 ± 266	Concave
**# 4**	1.34 ± 0.57	8	-307.3 ± 86.36	-34.27 ± 11.49	-345.52 ± 73.75	2.62 ± 1.11	1772 ± 244	Convex

E_{inter}, E_{vdw} and E_{elec} stand for intermolecular, van der Waals and electrostatic energy terms, respectively. E_{inter} is the sum of all energy contributions. BSA and AIRviol stand for Buried Surface Area and AIRs violations, respectively.

SUPPLEMENTARY FIGURE LEGENDS

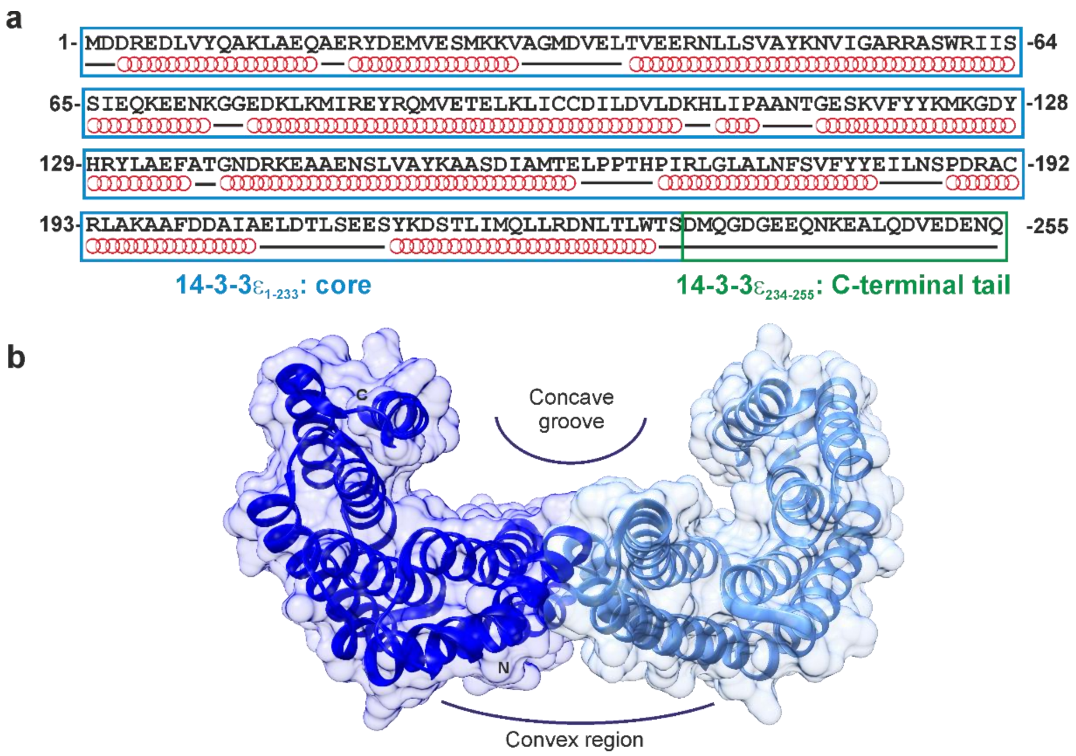

Figure S1. Structural organization of 14-3-3ε

(**a**) Amino acid sequence of 14-3-3ε, showing its secondary structure elements. The residues which form the structured core are marked into the blue squares. Green square corresponds to those residues which comprise the unstructured C-terminal tail. (**b**) Ribbon and surface representation of the crystallographic structure of the core of 14-3-3ε dimer (PDB: 2BR9) (ref. 1) with the monomers colored in blue and cyan.

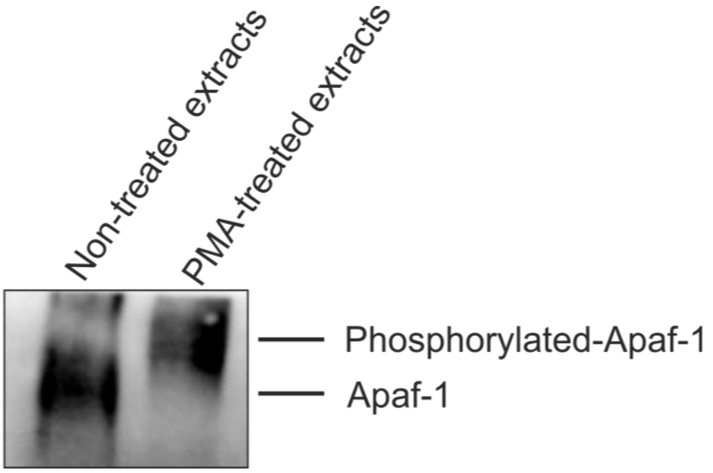

Figure S2. Detection of phosphorylated Apaf-1 upon PMA treatment.

Western-blot analysis of non-treated and PMA-treated lysates upon Apaf-1 immunoprecipitation, using a Phos-tag^{TM} SDS-PAGE and an anti-Apaf-1 antibody. Phosphorylated Apaf-1 showed a reduced electrophoretic mobility.

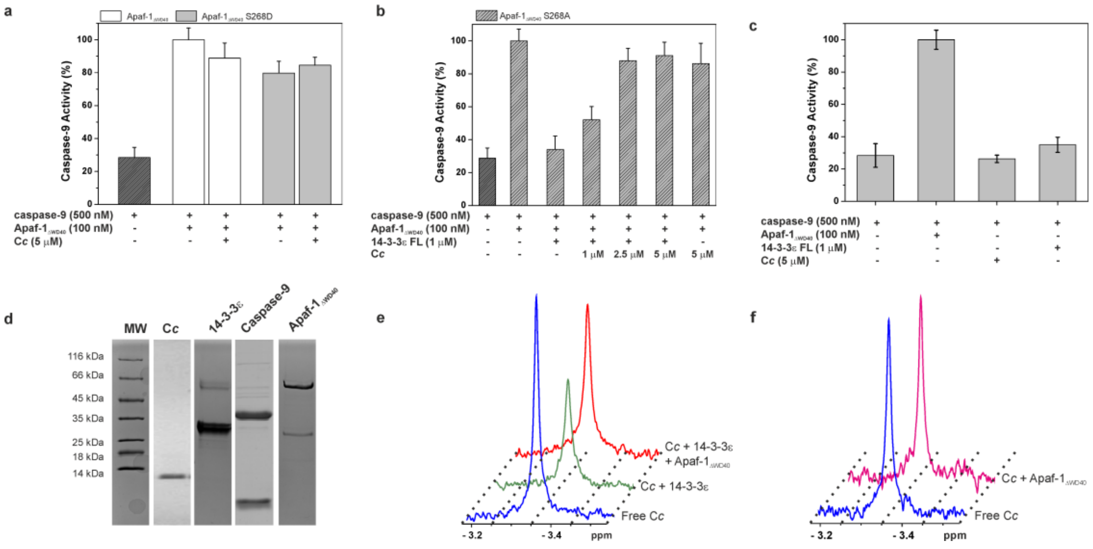

Figure S3. Controls experiments for the *in vitro* assays of caspase-9 activity

(**a**) Effect of C*c* on the caspase-9 activity triggered by Apaf-1$_{\Delta WD40}$ WT and the mutant S268D. (**b**) Apaf-1$_{\Delta WD40}$ S268A-mediated caspase-9 activity upon addition of 14-3-3ε FL at increasing concentrations of C*c*. (**c**) Effect of C*c* and 14-3-3ε on caspase-9 activity in the absence of Apaf-1$_{\Delta WD40}$. (**d**) SDS PAGE of the recombinant proteins used in caspase-9 activity assays. (**e, f**) 1D ^{1}H NMR spectra showing the Met-80 methyl signal of reduced C*c* upon successive additions of 14-3-3ε and Apaf-1$_{\Delta WD40}$ (**e**) or upon addition of Apaf-1$_{\Delta WD40}$ alone (**f**); protein concentration was 13 μM reduced C*c*, 13 μM 14-3-3ε and 5 μM Apaf-1$_{\Delta WD40}$.

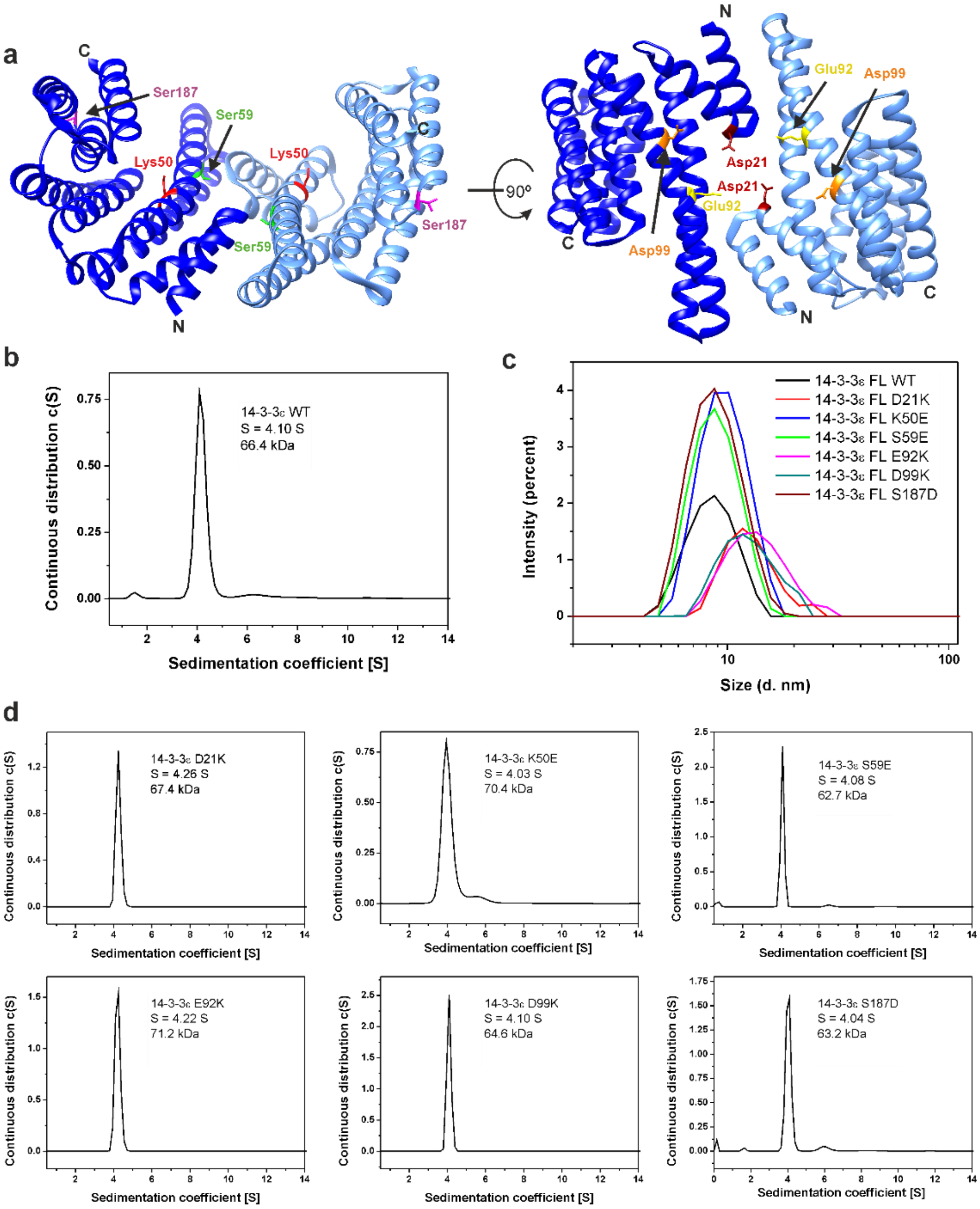

Figure S4. Dimerization state of 14-3-3ε species

(***a***) Structure of 14-3-3ε_{1-233} (PDB: 2BR9) highlighting residues Asp21 (dark red), Lys50 (red), Ser59 (green), Glu92 (yellow), Asp99 (orange) and Ser187 (purple). (**b**) Sedimentation velocity measurement of 14-3-3 FL WT. S is the sedimentation coefficient. (**c**) Particle size obtained by dynamic light scattering with 14-3-3ε FL species. (**d**) As panel B for mutant 14-3-3ε FL species.

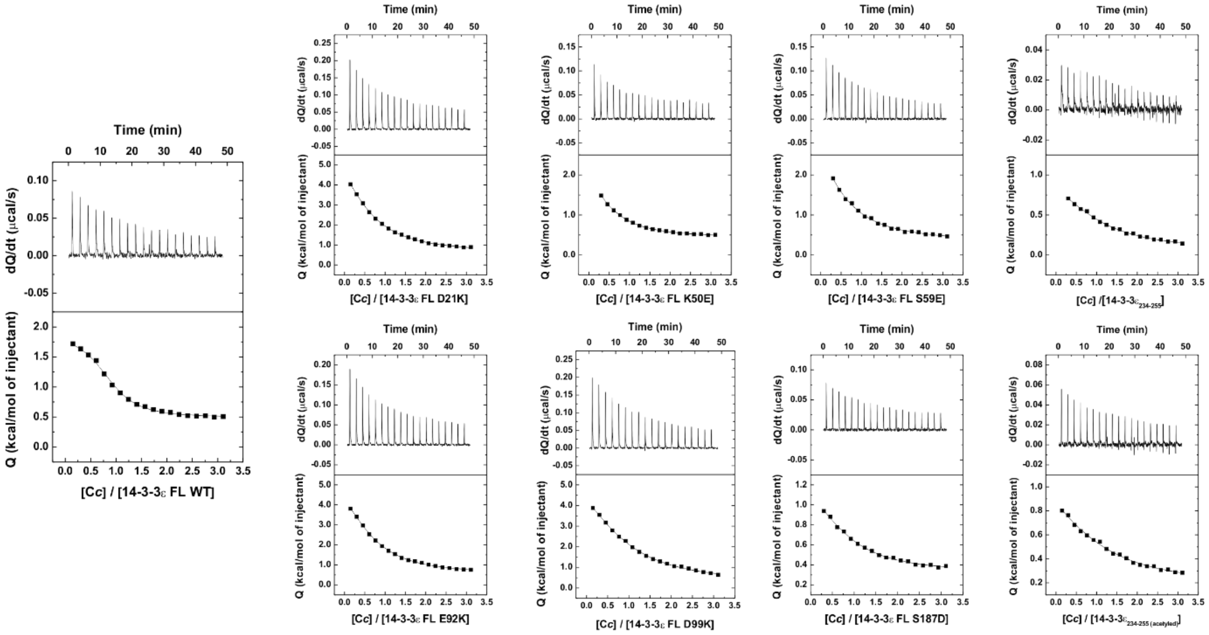

Figure S5. ITC binding assay of C*c* / 14-3-3ε FL complexes

The thermograms and binding isotherms (*top* and *bottom*, respectively) of reduced C*c* with WT and mutant species of 14-3-3ε are shown.

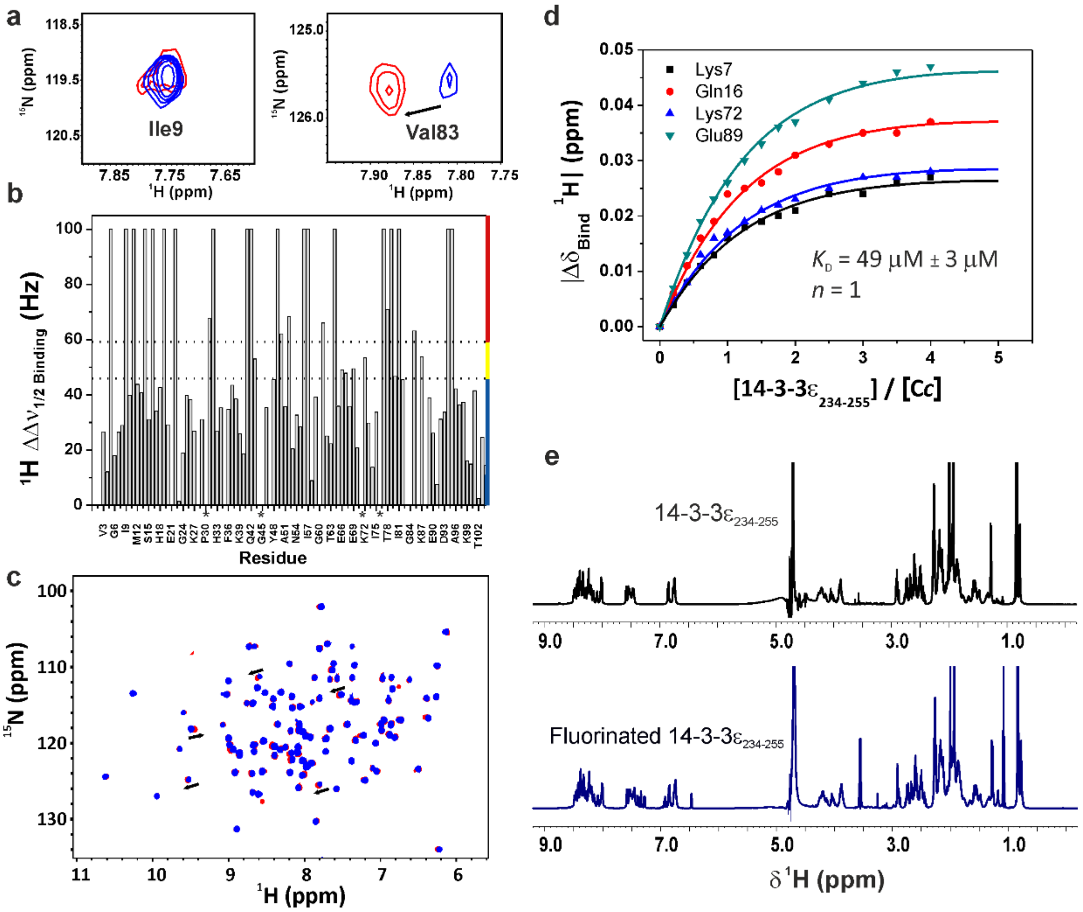

Figure S6. Binding of C*c* to 14-3-3ε constructs monitored by NMR

(**a**) Detail of superimposed 2D [^{1}H, ^{15}N] HSQC spectra of ^{15}N labelled C*c* (Fe^{2+}), either free (blue) or bound to 14-3-3ε FL in a C*c*:14-3-3ε ratio of 1:0.75 (red). (**b**) ^{1}H line-width differences (^{1}H $\Delta\Delta\nu_{½\ \mathrm{Binding}}$) between free and 14-3-3ε FL-bound C*c*. Resonance broaden beyond the threshold corresponding to the average plus 1-fold standard deviation (45.9 Hz) are in yellow. Signals with a line-width larger than the average value plus 2-fold standard deviation (59.2 Hz) are in red. Asterisks mark prolines. (**c**) Superimposed 2D [^{1}H, ^{15}N] HSQC spectra of ^{15}N-labeled C*c*, which is either free (blue) or bound to 14-3-3$\varepsilon_{234\text{-}255}$ at C*c*:14-3-3$\varepsilon_{234\text{-}255}$ molar ratio of 1:7. (**d**) Curves representing the best global fit of several amide signals of C*c* in direct dimension to a 1:1 14-3-3$\varepsilon_{234\text{-}255}$:C*c* binding model. (**e**) 1D ^{1}H NMR spectra of 14-3-3$\varepsilon_{234\text{-}255}$ before (*upper*) and after (*lower*) conjugation reaction with 5-fluorindole compound.

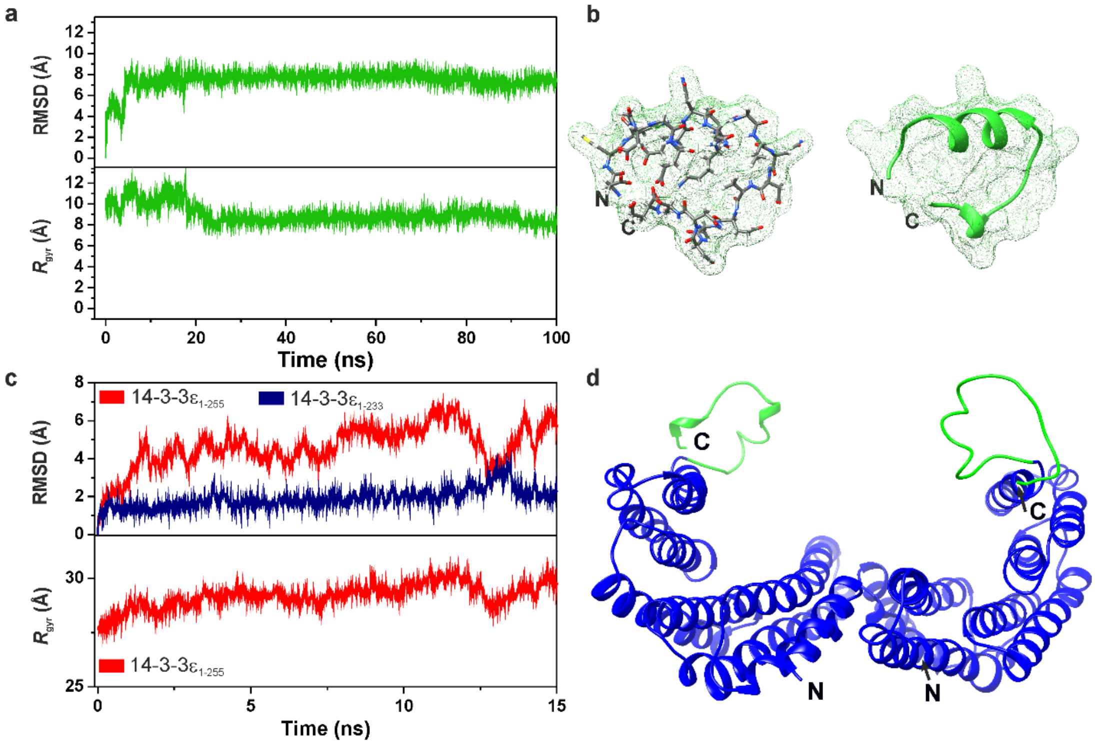

Figure S7. Molecular Dynamics analysis of 14-3-3$\varepsilon_{234-255}$ peptide and 14-3-3ε FL

(**a**) Analysis of the 100 ns MD trajectories of 14-3-3$\varepsilon_{234-255}$. The time evolution of the backbone RMSD values and the radius of gyration (R_{gyr}) are represented in *upper* and *lower* panel, respectively. (**b**) Representation of the closest structure to the average one from the last 40 ns of the computation. Representation as atoms and bonds is in the *left* panel and ribbon representation is in the *right* panel. (**c**) Analysis of the trajectories from MD of 14-3-3ε FL (14-3-3ε_{1-255}) protein. Backbone RMSD values along the trajectories of 14-3-3ε FL and 14-3-3ε *core* (14-3-3ε_{1-233}) are represented in red and blue, respectively. The time evolution of the backbone R_{gyr} values for 14-3-3ε FL is represented in red (*lower* panel). (**d**) The closest structure to the average one obtained from the last 5 ns of the computation. The resulting model is represented in blue with the 14-3-3$\varepsilon_{234-255}$ tail colored in green.

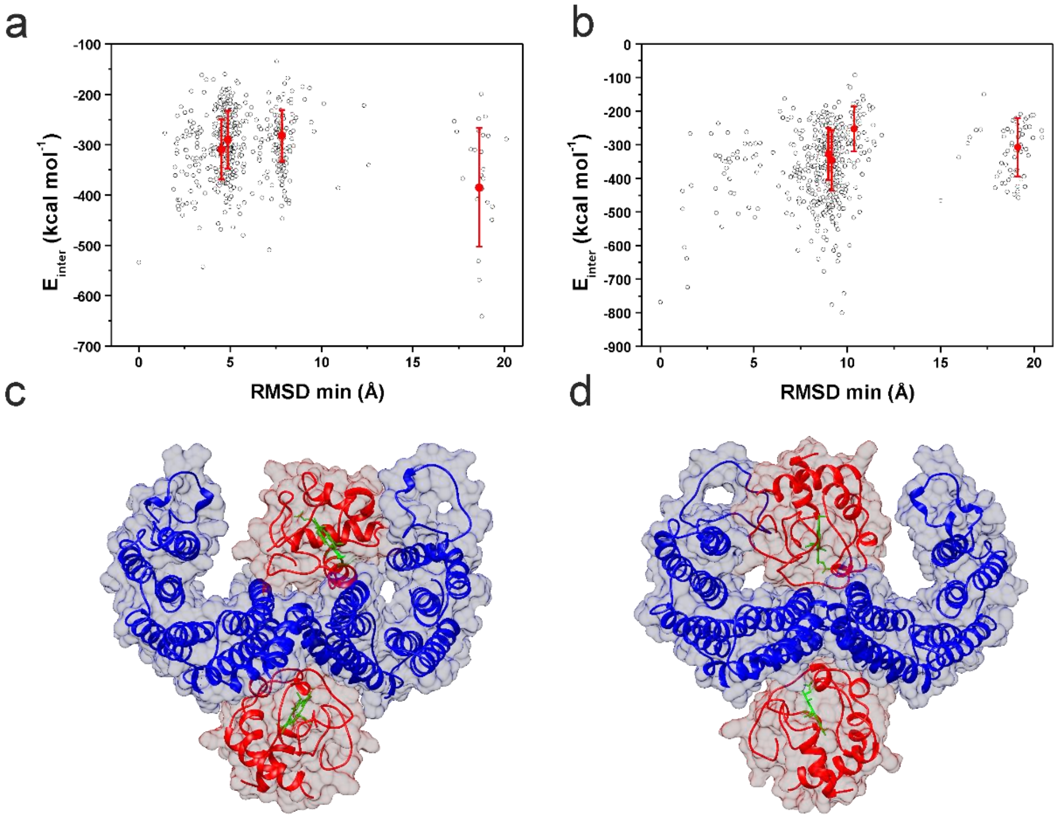

Figure S8. HADDOCK molecular docking of C*c* and 14-3-3ε FL without (*left* panels) or with (*right* panels) Lys244 from 14-3-3ε included in the calculations as an active residue

(**a, b**) E_{inter} values represented as a function of their RMSD values. White dots correspond to the individual structures and red dots correspond to the cluster averages with the standard deviation indicated by bars. (**c, d**) Transparent surfaces, along with ribbons, of the best two complex models with minimal E_{inter} at either the concave or convex binding site of 14-3-3ε. 14-3-3ε FL is represented in blue, C*c* in red and heme group is colored in green.

SUPPLEMENTARY REFERENCES

1. Yang, X. et al. Structural basis for protein-protein interactions in the 14-3-3 protein family. *Proc Natl Acad Sci USA* **103**, 17237-17242 (2006).
